# Changes in higher order aberrations after central corneal regularization - a comparative two-year analysis of a semi-automated topography-guided photorefractive keratectomy combined with corneal cross-linking

**DOI:** 10.1186/s40662-020-00179-2

**Published:** 2020-03-03

**Authors:** Katja C. Iselin, Philipp B. Baenninger, Lucas M. Bachmann, Frank Bochmann, Michael A. Thiel, Claude Kaufmann

**Affiliations:** 1 Department of Ophthalmology, Lucerne Cantonal Hospital, Lucerne, Switzerland; 2 Medignition Healthcare Innovations, Zurich, Switzerland

**Keywords:** Keratoconus, Corneal cross-linking, CXL-plus, Photorefractive keratectomy, Central corneal regularization, Higher order aberrations, Coma-like aberrations

## Abstract

**Background:**

The optical quality in progressive keratoconus deteriorates due to ectasia and distortion of the corneal shape and optics. While corneal cross-linking (CXL) aims at stopping disease progression, “CXL-Plus” combines CXL with excimer laser ablation to improve visual function. Central Corneal Regularization (CCR) represents a therapeutic excimer laser modality specifically designed to smoothen the ectatic corneal shape and to reduce higher order aberrations (HOA). We set out to compare CXL-Plus, consisting of CXL combined with CCR, with CXL by itself for patients with progressive keratoconus.

**Methods:**

Retrospective 2-year matched group analysis of patients who either underwent CXL-Plus (*n* = 28) or CXL as a sole procedure (*n* = 28) for progressive keratoconus. Main outcome parameters were HOA, visual function and tomographic results 12 and 24 months postoperatively.

**Results:**

After 12 months, the total HOA root mean square wavefront error was reduced from 0.79 ± 0.30 to 0.40 ± 0.19 μm (CXL-Plus; *p* <  0.0001) and changed from 0.71 ± 0.28 to 0.73 ± 0.36 μm (CXL; *p* = 0.814). Uncorrected distance visual acuity improved from 0.70 ± 0.35 to 0.36 ± 0.29 logMAR (CXL-Plus; *p* = 0.0002) and from 0.65 ± 0.39 to 0.46 ± 0.37 logMAR (CXL; *p* = 0.067), translating to gains of three or more lines in 50% (CXL-Plus) and 36% (CXL) of patients. The steepest keratometry value (Kmax) regressed by 5.84 D (CXL-Plus; *p* <  0.0001) and 0.66 D (CXL; *p* = 0.752). For none of the investigated parameters a statistically significant change could be shown between 12 and 24 months.

**Conclusions:**

CXL-Plus in the form of a CCR reduces HOA and Kmax more effectively than CXL as a sole procedure.

## Background

Corneal irregularities in ectatic diseases such as keratoconus degrade the eye’s optical quality and visual performance because of the increase in higher order aberrations (HOA) [1]. Although the primary aim of corneal cross-linking (CXL) is to stop the progression of keratoconus ectasia, it may, as a welcomed side effect, also result in a decrease in corneal curvature and in a flattening of the apex. This often leads to improvements in uncorrected distance visual acuity (UDVA) and best spectacle-corrected visual acuity (BSCVA). Recent reviews suggest that visual and topographic outcomes can be further improved by combining CXL with simultaneous photorefractive keratectomy (PRK) to reshape the apex. This is also termed CXL-Plus, i.e. ectasia management plus refractive treatment [2–4]. Surprisingly, little is known about the effect of CXL-Plus on HOA: Just a single study measured the changes in higher-order aberrations [5], but without distinguishing between the aberrations of the anterior and posterior corneal surface. It is also noteworthy that most studies on CXL-Plus, apart from a few exceptions [5–7], do not compare their results with a control group that received CXL as a sole procedure.

Central corneal regularization (CCR) is a novel, customized, topography-guided, transepithelial PRK treatment specifically developed for combined use with CXL (iVIS™ platform, Ligi Tecnologie Medicali S.r.l.). CCR is semi-automated in the sense that it involves a point-and-click operation in the planning software that calculates an optimized excimer ablation with the greatest possible regularization of the cone tip along with the lowest possible tissue consumption.

We therefore set out to analyze the effect of combined CCR-CXL treatments on HOA, visual function and tomographic results and to compare the results with the outcome after CXL alone.

## Patients and methods

### Study design

This single-center, retrospective, 2-year, matched group analysis at the Lucerne Cantonal Hospital compared patients who underwent combined CCR-CXL (hereinafter termed CXL-Plus) to patients who underwent CXL alone during the same period.

### Patients

The diagnosis of keratoconus was based on Pentacam® HR elevation data and on pachymetry maps (Oculus GmbH). Keratoconus patients with a minimal corneal thickness of 400 μm were offered CXL in case of confirmed disease progression. Keratoconus progression was defined as consistent change in at least one of the two following parameters during the previous 12 months: an increase in the steepest keratometry value (Kmax) of ≥1 D [8–12] and/or a decrease in minimal corneal thickness of ≥10 μm [13, 14].

Simultaneous CCR was offered to patients presenting with a minimal corneal thickness of 450 μm or more, i.e. to patients who presumably had enough stromal tissue to meet the 400 μm pachymetry threshold even in combination with excimer ablation. A tomographic proof of progression was not performed in patients who already presented with a corneal thickness of just over 400 μm at the first consultation and who reported a current ongoing visual deterioration [8, 10, 14, 15]: These cases were offered cross-linking because there was a risk that the required minimum corneal thickness of 400 μm would soon be undershot.

Standardized examinations were performed at baseline, at 1, 3, and 12 months after the procedure(s), and, optionally, after 24 months. Examinations included the assessment of UDVA, BSCVA, slit-lamp biomicroscopy, and Pentacam® HR Scheimpflug imaging 12 and 24 months postoperatively to evaluate tomographic indices, aberrometric and densitometric values. For the Pentacam® images, patients were asked to blink repeatedly and to keep their eyes wide open during the exposure. The scans were centered on the pupil and only accepted if the recording quality was rated “OK” by the software. For the computation of the aberrations in the Pentacam® software, a pupil diameter of 3.5 mm, a refractive index of 1.3375, and the standard of the Optical Society of America for the representation of Zernike polynomials were used [16]. The root mean square (RMS) wave front error values were calculated for total aberrations up to the sixth order, lower order aberrations, HOA (third to the sixth order), and coma-like aberrations (sum of squared coefficients of Z_3_^− 1^, Z_3_^+ 1^, Z_5_^− 1^, and Z_5_^+ 1^) for the entire cornea and separately for the anterior and posterior corneal surface. The RMS values are equivalent to the standard deviations of the wavefront errors, providing a convenient overview of the relative amount of the eyes’ aberration [17]. The densitometry values were calculated for total corneal thickness within the central 2 mm zone and the adjacent annulus extending from 2 mm to 6 mm. The output values are expressed in grayscale units with “0″ indicating minimum light scatter (maximum transparency) and “100″ maximum light scatter (minimum transparency) [18].

### Surgical techniques

The concept of CCR and the iVIS Suite™ platform modules are described in detail elsewhere [19]. In brief, a PrecisioHD™ Scheimpflug tomographer was used to acquire corneal elevation maps, each consisting of 39,000 measurement points with an auto-validated repeatability of ≤3 μm [20]. Customized transepithelial surface ablations were planned in the CCR mode of the CIPTA™ (Corneal Interactive Programmed Topographic Ablation) software [21]. In contrast to purely refractive ablations, which have large optical zones and relatively small transition zones, regularizing the ectatic corneal shape by CCR consists of a small (0.1 to 1.5 mm) optical zone centered on the apex and a very large transition zone, both of which are individually determined by the software. The transition zone in CCR represents a pure topography-guided custom ablation with gradually decreasing power, which creates an optically active blend zone, making it possible to use a very small optical zone that restricts the ablation to a few tens of a micron. The calculation of the transition zone is based on the identification of the most critical hemi-meridian, in which the highest change in refractive power occurs between the optical zone and the untreated periphery. The calculation routine attempts to limit the change in refractive power in this hemi-meridian to a value of 80 D or as close to 80 D as possible. The remaining transition zone is then modelled by aiming for the same power change for each further hemi-meridian. The individualization of the transition zone allows for each hemi-meridian a continuity of refractive power between optical zone and transition zone and a constant radial slope within the transition zone itself in order to reduce aberrations as best as possible and to achieve a re-epithelialization that is as uniform as feasible. The regularization proposed by the routine corresponds to an optimization of the individual preconditions. In the standard settings, the calculation routine aims at the greatest possible reduction of the corneal cylinder as well as at the asphericity of a prolate aspheric, which is 0.5 D steeper in the center than in the peripheral 6 mm zone. These corrections induce a spherical shift which is compensated by the spherical part of the ablation. However, the diameter of the optical zone and the maximum refractive power changes can still be adjusted by the surgeon. Treatment planning also included complimentary epithelial removal within the central 9.0 mm to facilitate subsequent CXL. CCR was performed by a 1000-Hz iRes™ laser with a spot size of 0.65 mm. After excimer ablation, topical mitomycin C 0.02% was applied for 30 s using a soaked sponge. CCR was followed by CXL, which was identical in both groups except for the epithelial removal technique (by excimer ablation or by alcohol debridement; additional file 1). Isotonic riboflavin solution (Peschke M®, Peschke Meditrade GmbH) was applied if ultrasonic pachymetry confirmed a minimal corneal thickness of 400 μm. Hypotonic solution (Peschke H®, Peschke Meditrade GmbH) was used if the pachymetry readings were below 400 μm. Postoperative treatment consisted of a bandage contact lens, topical ofloxacin 0.3% (Floxal UD, Bausch & Lomb Swiss AG), and topical dexamethasone 0.1% (Dexafree UD, Théa Pharma SA) q.i.d. After complete re-epithelialization, the contact lens was removed, antibiotics were discontinued, and steroids were gradually reduced over 1 month.

### Data selection

We aimed to analyze patients who were treated for progressive keratoconus with uniform irradiation schemes who did not have any other eye conditions that could influence their visual acuity or the course of the disease. Accordingly, the exclusion criteria were defined as follows: indications for CXL other than keratoconus, a history of previous eye surgery or any other vision-threatening ocular disease, any systemic disease, pregnancy or current medications with potential ocular side effects, the inability to comply with visual acuity measurements and refraction, age > 45 years [10], incomplete data sets (refraction, slit lamp biomicroscopy, tomography), and follow-up periods shorter than 12 months.

A review of combined procedures, from the most recent to the earliest, identified 204 CXL-Plus procedures. After applying the exclusion criteria (additional file 2), there were 96 potential study patients in the CXL-Plus group. Next, a control group of patients who underwent CXL as a sole procedure during the same time period was identified using the same inclusion and exclusion criteria. Going back in time from the most recent to earlier CXL treatments, the study included the first 96 patients with complete data sets.

Sample size consideration were made on the basis of HOA changes between groups from baseline to the 12-month follow-up. We assumed to find a change of 0.1 with a standard deviation of 0.4 in the CLX alone group and a change of 0.4 with a standard deviation of 0.4 in the CLX Plus group. When choosing an alpha error of 5% and a power of 90%, the corresponding number of subjects per group would be 28. Therefore, as a final step, the study population was reduced to two groups which showed no statistically significant difference in relevant baseline parameters (UDVA, BSCVA, mean refractive spherical equivalent MRSE, corneal thickness, topographic cylinder, Kmax, and HOA): using a probability matching approach, we selected 28 patients per group based on the estimated probability for being member of the combined group of 50 to 80%. The matched groups included 28 patients each at baseline and after 12 months. After 24 months, the CXL-Plus group consisted of 16 patients and the CXL group of 10 patients.

### Analysis

#### Descriptive statistics and analysis

Interval scaled variables were summarized as means and standard deviations. Dichotomous variables were described as ratios and percentages. Visual acuity measurements were converted from lines (Snellen) into logMAR units. Changes in UDVA and BSCVA were assessed using bar graphs, which only included eyes with visual acuity ≥0.01. Visual improvement was defined as ≥2 lines (Snellen) difference between follow-up visits.

#### Statistical analysis

The paired t test (within group comparison) was performed to compare the postoperative outcomes with the baseline values and to analyze changes in outcomes from 12 to 24 months postoperatively. The unpaired t test (between group comparisons) was performed to compare outcome data at 12 and 24 months postoperatively between the groups.

Statistical analysis was performed using the Stata 14.2 statistics software package (StataCorp, College Station, TX).

## Results

The excimer ablations in the CXL-Plus group were characterized by the following parameters (all values as mean values ± standard deviations): The diameter of the optical zone was 1.61 ± 0.37 mm, that of the transition zone 7.12 ± 1.34 mm. The ablated stromal volume amounted to 0.36 ± 0.15 mm^3^, the total volume (stroma plus all ablated epithelium within the 9 mm zone) amounted to 3.98 ± 0.92 mm^3^. The ablation depth was 45 ± 11 μm, the calculated minimum corneal thickness after ablation was 435 ± 25 μm. The minimum corneal thickness measured by ultrasound pachymetry at the beginning of the subsequent CXL (before riboflavin application) was 436 ± 30 μm and 471 ± 20 μm at the end of CXL (after UV irradiation), wherein in half of the cases (14 of 28) a hypotonic riboflavin solution to swell the stroma was used at least once. In the CXL-only group, the corresponding ultrasonic pachymetry values were 434 ± 24 μm before and 476 ± 19 μm after irradiation and in 18 of 28 cases the hypotonic riboflavin solution was used at least once.

The visual, refractive, tomographic, aberrometric, and densitometric outcomes at baseline and after 12 months are summarized in Table 1, the results after 24 months in Table 2. As an intended consequence of the matching process during data selection, there was no statistically significant difference between the groups regarding the baseline parameters (Table 1). After 12 months, total and anterior corneal surface HOA were significantly reduced in the CXL-Plus group, while the aberrations in the CXL group did not change significantly. Also, no significant changes were documented in the aberrations of the posterior corneal surface in either group. Kmax regressed significantly by 5.84 D in the CXL-Plus group and non-significantly by 0.66 D in the CXL group. The densitometric values within the central 0–2 and 2–6 mm increased significantly in both groups with no significant difference between the groups. The UDVA improvement of 0.34 logMAR in the CXL-Plus group was significant, while the 0.19 logMAR improvement in the CXL group was only very close to statistical significance. In contrast, BSCVA significantly improved in both groups.

**Table 1:** Visual, refractive, tomographic, aberrometric, and densitometric parameters at baseline and 12 months postoperatively in eyes treated with central corneal regularization combined with corneal cross-linking and in eyes treated with corneal cross-linking as a single procedure

	CXL-Plus group (*n* = 28)	CXL group (n = 28)	*p*-value (CXL-Plus vs. CXL 0)	*p*1 (CXL-Plus 0 vs. 12)	CXL-Plus group (n = 28)	CXL group (n = 28)	*p*1 (CXL 0 vs. 12)	*p*2 (CXL-Plus vs. CXL 12)
	Baseline				12 months follow-up			
	**Mean (SD)**	**Mean (SD)**			**Mean (SD)**	**Mean (SD)**		
**Sex***(% female)*	28.57%	14.29%	0.329					
**Age***(years)*	27.07 (9.14)	25.82 (9.25)	0.613					
**UDVA***(logMAR)*	0.70 (0.35)	0.65 (0.39)	0.616	0.0002	0.36 (0.29)	0.46 (0.37)	0.067	0.265
**BSCVA***(logMAR)*	0.23 (0.19)	0.20 (0.18)	0.547	0.006	0.10 (0.15)	0.06 (0.11)	0.0009	0.260
**Refractive values**								
*MRSE (D)*	−1.96 (2.76)	−0.92 (1.80)	0.101	0.023	−0.54 (1.63)	−0.05 (1.87)	0.082	0.301
*Manifest Sphere (D)*	−0.43 (2.34)	0.37 (2.08)	0.182	0.118	0.41 (1.53)	1.65 (1.90)	0.020	0.010
*Manifest Cylinder (D)*	−3.06 (2.24)	−2.57 (2.49)	0.442	0.020	−1.91 (1.17)	−3.40 (2.11)	0.184	0.002
**Corneal Thickness** (*μm)*	478.82 (20.42)	470.36 (17.03)	0.098					
**Curvature Values**								
*Cylinder (D)*	3.84 (1.79)	3.64 (1.88)	0.685	0.240	3.24 (1.98)	4.03 (2.22)	0.481	0.166
*K1 (D)*	43.88 (3.19)	44.68 (1.62)	0.242	0.006	41.64 (2.67)	43.20 (2.29)	0.007	0.023
*K2 (D)*	47.71 (3.79)	48.32 (2.51)	0.481	0.006	44.88 (3.63)	47.23 (3.08)	0.152	0.012
*Kmax (D)*	54.41 (5.22)	54.36 (3.74)	0.967	< 0.0001	48.57 (4.49)	53.70 (4.52)	0.554	0.0001
**Aberrometric Values**								
*Cornea Total*								
*RMS total (μm)*	2.95 (1.03)	2.62 (0.97)	0.223	< 0.0001	1.62 (0.82)	2.72 (1.35)	0.752	0.0005
*RMS LOA (μm)*	2.84 (0.99)	2.52 (0.94)	0.220	< 0.0001	1.57 (0.80)	2.61 (1.31)	0.769	0.0007
*RMS HOA (μm)*	0.79 (0.30)	0.71 (0.28)	0.307	< 0.0001	0.40 (0.19)	0.73 (0.36)	0.817	0.0001
*RMS Coma-like (μm)*	0.75 (0.29)	0.68 (0.28)	0.364	< 0.0001	0.31 (0.21)	0.65 (0.36)	0.648	< 0.0001
*Anterior corneal surface*								
*RMS total (μm)*	3.30 (1.17)	2.97 (1.06)	0.274	< 0.0001	1.70 (0.93)	2.98 (1.34)	0.975	0.0001
*RMS LOA (μm)*	3.18 (1.12)	2.85 (1.03)	0.256	< 0.0001	1.65 (0.92)	2.88 (1.29)	0.924	0.0001
*RMS HOA (μm)*	0.90 (0.34)	0.81 (0.30)	0.298	< 0.0001	0.40 (0.21)	0.79 (0.36)	0.822	< 0.0001
*RMS Coma-like (μm)*	0.85 (0.33)	0.76 (0.30)	0.316	< 0.0001	0.32 (0.22)	0.72 (0.36)	0.417	< 0.0001
*Posterior corneal surface*								
*RMS total (μm)*	0.80 (0.31)	0.75 (0.26)	0.516	0.692	0.83 (0.25)	0.81 (0.29)	0.419	0.783
*RMS LOA (μm)*	0.76 (0.29)	0.72 (0.26)	0.589	0.675	0.79 (0.24)	0.77 (0.28)	0.492	0.775
*RMS HOA (μm)*	0.23 (0.09)	0.22 (0.07)	0.527	0.662	0.24 (0.08)	0.23 (0.09)	0.644	0.662
*RMS Coma-like (μm)*	0.21 (0.09)	0.19 (0.07)	0.417	0.489	0.23 (0.14)	0.20 (0.08)	0.318	0.350
**Densitometric Values**								
*0.0–2.0 mm*	19.00 (1.46)	18.47 (2.04)	0.269	< 0.0001	24.49 (5.43)	25.70 (6.89)	< 0.0001	0.469
*2.0–6.0 mm*	16.83 (1.08)	16.41 (2.39)	0.401	0.001	18.53 (2.40)	18.69 (2.65)	0.001	0.814

*BSCVA*= best spectacle-corrected visual acuity; *coma-like*= primary and secondary coma aberration; *CXL=* corneal cross-linking; *CXL*-Plus= central corneal regularization combined with CXL; *HOA=* higher order aberrations; *K1=* flattest meridian, *K2=* steepest meridian, *Kmax=* steepest radius of anterior curvature; *LOA=* lower order aberrations; *MRSE=* mean refractive spherical equivalent; *RMS=* root mean square; *UDVA=* uncorrected distance visual acuity. The results are expressed as means (standard deviation). The level of statistical significance was set at *p* ≤ 0.05. *P*-value = the difference between CXL-Plus and CXL values at baseline. *p*1 = the difference within a group between baseline values and postoperative values at 12 months. *p*2 = the difference between CXL-Plus values and CXL alone values 12 months postoperatively

**Table 2:** Visual, refractive, tomographic, aberrometric, and densitometric parameters 12 months and 24 months postoperatively in eyes treated with central corneal regularization combined with corneal cross-linking and in eyes treated with corneal cross-linking as a single procedure

	CXL-Plus group (n = 28)	CXL group (*n* = 28)	CXL-Plus group (*n* = 16)	CXL group (*n* = 10)	*p*1 (CXL-Plus group 12 vs. 24)*	*p*2 (CXL 12 vs. 24)*	*p*3 (CXL-Plus vs. CXL 24)
	12 months follow-up		24 months follow-up				
	Mean (SD)	Mean (SD)	Mean (SD)	Mean (SD)			
**UDVA***(logMAR)*	0.36 (0.29)	0.46 (0.37)	0.53 (0.51)	0.29 (0.28)	0.570	0.201	0.187
**BSCVA***(logMAR)*	0.10 (0.15)	0.06 (0.11)	0.10 (0.14)	0.01 (0.07)	0.072	0.879	0.072
**Refractive values**							
*MRSE (D)*	−0.54 (1.63)	− 0.05 (1.87)	−1.30 (3.15)	0.66 (2.23)	0.464	0.586	0.100
*Manifest Sphere (D)*	0.41 (1.53)	1.65 (1.90)	0.02 (2.58)	2.03 (2.20)	0.733	0.548	0.053
*Manifest Cylinder (D)*	−1.91 (1.17)	−3.40 (2.11)	−2.63 (1.87)	− 2.73 (1.60)	0.051	0.758	0.890
**Curvature Values**							
*Cylinder (D)*	3.24 (1.98)	4.03 (2.22)	3.85 (2.49)	4.56 (1.92)	0.451	0.945	0.450
*K1 (D)*	41.64 (2.67)	43.20 (2.29)	41.00 (3.09)	42.77 (1.43)	0.061	0.162	0.104
*K2 (D)*	44.88 (3.63)	47.23 (3.08)	44.85 (4.81)	47.33 (2.43)	0.155	0.080	0.145
*Kmax (D)*	48.57 (4.49)	53.70 (4.52)	49.69 (5.42)	53.00 (4.94)	0.796	0.023	0.131
**Aberrometric Values**							
*Cornea Total*							
*RMS total (μm)*	1.62 (0.82)	2.72 (1.35)	2.22 (1.15)	2.81 (1.52)	0.124	0.860	0.272
*RMS LOA (*μm*)*	1.57 (0.80)	2.61 (1.31)	2.15 (1.12)	2.72 (1.46)	0.123	0.911	0.272
*RMS HOA (μm)*	0.40 (0.19)	0.73 (0.36)	0.53 (0.29)	0.70 (0.43)	0.191	0.228	0.239
*RMS Coma-like (μm)*	0.31 (0.21)	0.65 (0.36)	0.46 (0.32)	0.57 (0.47)	0.147	0.087	0.508
*Anterior corneal surface*							
*RMS total (μm)*	1.70 (0.93)	2.98 (1.34)	2.23 (1.15)	2.96 (1.51)	0.181	0.303	0.175
*RMS LOA (μm)*	1.65 (0.92)	2.88 (1.29)	2.17 (1.12)	2.86 (1.45)	0.177	0.311	0.185
*RMS HOA (μm)*	0.40 (0.21)	0.79 (0.36)	0.51 (0.27)	0.74 (0.43)	0.338	0.289	0.105
*RMS Coma-like (μm)*	0.32 (0.22)	0.72 (0.36)	0.47 (0.29)	0.66 (0.44)	0.203	0.185	0.171
*Posterior corneal surface*							
*RMS total (μm)*	0.83 (0.25)	0.81 (0.29)	0.91 (0.26)	0.82 (0.29)	0.608	0.871	0.419
*RMS LOA (μm)*	0.79 (0.24)	0.77 (0.28)	0.87 (0.25)	0.78 (0.28)	0.523	0.918	0.403
*RMS HOA (μm)*	0.24 (0.08)	0.23 (0.09)	0.26 (0.08)	0.23 (0.09	0.343	0.894	0.384
*RMS Coma-like (μm)*	0.23 (0.14)	0.20 (0.08)	0.22 (0.14)	0.19 (0.10)	0.453	0.885	0.579
**Densitometric Values**							
*0.0–2.0 mm*	24.49 (5.43)	25.70 (6.89)	26.04 (8.89)	26.88 (8.52)	0.700	0.826	0.814
*2.0–6.0 mm*	18.53 (2.40)	18.69 (2.65)	19.03 (4.08)	18.71 (3.54)	0.807	0.432	0.840

* based on patients providing data to both time points

*BSCVA=* best spectacle-corrected visual acuity; *Coma-like*= primary and secondary coma aberration; *CXL=* corneal cross-linking; *CXL*-Plus= central corneal regularization combined with CXL; *HOA=* higher order aberrations; *K1* flattest meridian, *K2=* steepest meridian, *Kmax=* steepest radius of anterior curvature; *LOA=* lower order aberrations; *MRSE=* mean refractive spherical equivalent; *RMS=* root mean square; *UDVA=* uncorrected distance visual acuity. The results are expressed as means (standard deviation). The level of statistical significance was set at *p* = ≤; 0.05. p1 = the difference within the CXL-Plus group between postoperative values at 12 and 24 months postoperatively, *p*2 = the difference within the CXL-Plus group between postoperative values at 12 and 24 months postoperatively, *p*3 = the difference between CXL-Plus values and CXL alone values 24 months postoperatively

No significant change was observed between the 12- and 24-month follow-up for all parameters within and between the two groups (Table 2).

**Fig. 1:**
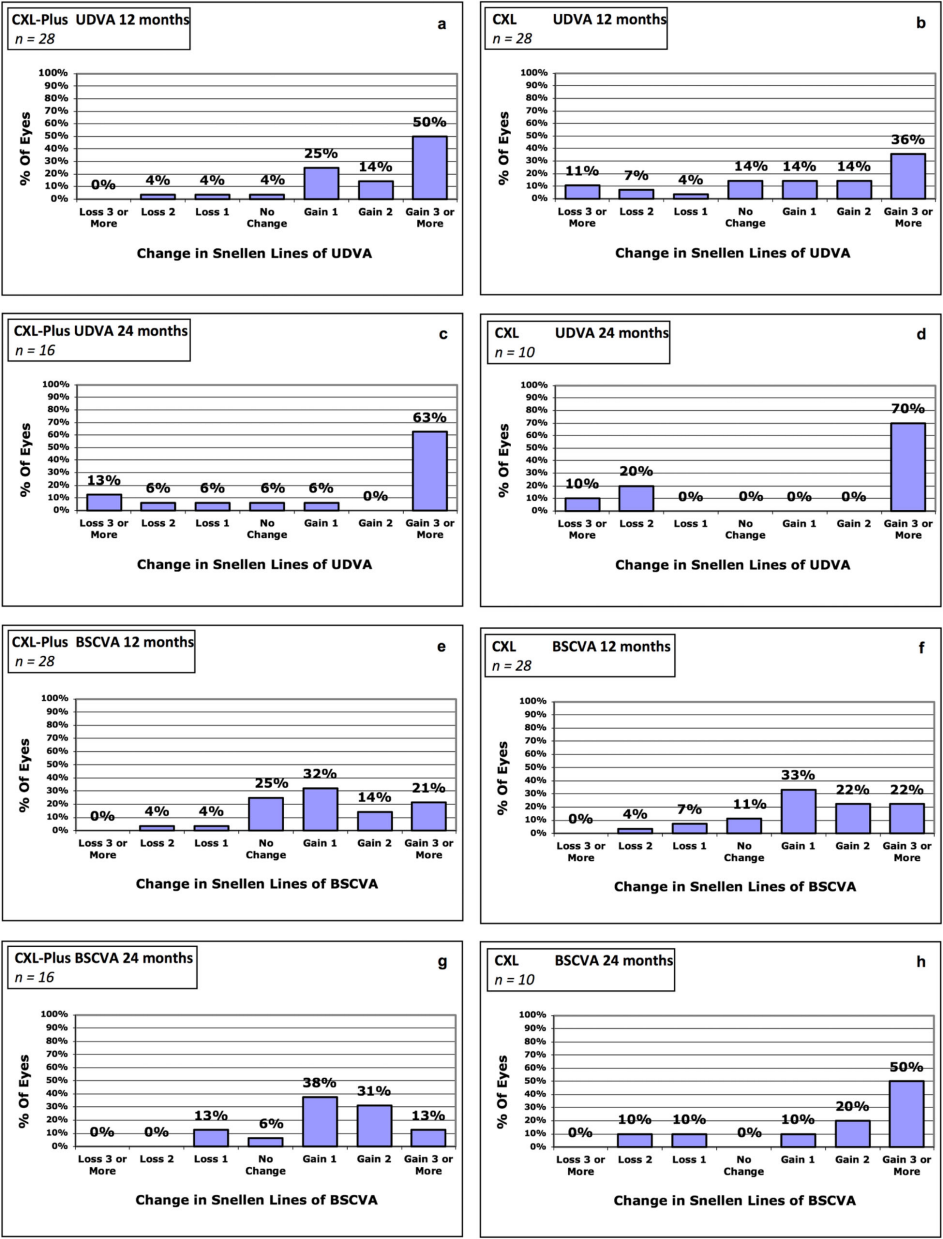
The changes in uncorrected distance visual acuity (UDVA) and best spectacle-corrected visual acuity (BSCVA) shown as percentages of lines gained/lines lost. Changes in UDVA after 12 months in the CXL-Plus group (**a**) and in the CXL group (**b**) and after 24 months in the CXL-Plus group (**c**) and in the CXL group (**d**) respectively. Changes in BSCVA after 12 months in the CXL-Plus group (**e**) and in the CXL group (**f**) and after 24 months in the CXL-Plus group (**g**), and in the CXL group (**h**) respectively. CXL = Corneal cross-linking, CXL-Plus = Central corneal regularization combined with CXL

Figure 1 shows the Snellen line changes in UDVA and BSCVA from baseline to the postoperative follow-ups for both groups. After 12 months of follow-up (24-month values in parentheses), 64% (63%) of the eyes in the CXL-Plus group and 50% (70%) of the eyes in the CXL group gained 2 or more lines in UDVA, while 4% (19%) of the eyes in the CXL-Plus group and 18% (30%) of the eyes in the CXL group lost 2 or more lines. The differences between the groups were less pronounced with regard to BSCVA: in the CXL-Plus group, 35% (44%) of the eyes showed gains of 2 or more lines, while 4% (0%) showed losses of 2 or more lines; in the CXL group, 44% (70%) showed gains, while 4% (10%) showed losses.

## Discussion

The main finding of this study was that CXL-Plus was compared favorably with CXL as a sole procedure with regard to the reduction of HOA after 12 months. The topography-guided keratectomy by CCR regularized the ectatic corneal shape significantly, as indicated by the reduced RMS values: Since the anterior corneal surface as the main source of corneal HOA [22] represented also the target of excimer regularization, the RMS values for the anterior corneal surface and the total cornea changed in the same sense. The HOA of the posterior corneal surface in both groups were not significantly affected. This is plausible against the background that the direct laser effect is limited to the surface and the CXL effect is concentrated on the anterior 200 to 300 μm [23]. However, altered biomechanical properties of the cornea have been held responsible for the fact that the aberrations of the posterior surface may change after excimer laser surgery, and for the same reason changed aberrations of the posterior surface after CXL have been considered conceivable [1]. The proportion of coma-like aberrations in the total HOA was 85% on average. This confirms earlier reports according to which coma-like aberrations represent the dominant aberration mode in keratoconic eyes, [24] most likely because of the asymmetric corneal power distribution due to the apex displacement [25].

The changes in corneal shape (Kmax) in the two groups corresponded to the changes in aberrations: the flattening of the steepest keratometric value was highly significant in the CXL-Plus group but not in the CXL group. The objectively collected aberrometry data also found their counterpart in the refraction, which is associated with less preciseness due to its partly subjective nature: MRSE values improved in both groups, but only in the CXL-Plus group did the change reach statistical significance.

With regard to visual acuity, the UDVA in the combined group improved significantly whereas in the CXL Group, the gain in UDVA was approaching but not reaching statistical significance.

BSCVA, on the other hand, increased significantly in both groups and the extent of the improvement was very similar. This is surprising for two reasons. On the one hand, we would expect a higher BSCVA improvement in the combined group, since the regularization of the corneal anterior surface has reduced the relative proportion of the optical error that cannot be corrected by sphere and cylinder. On the other hand, the significant increase in BSCVA in the CXL group cannot be attributed to equally pronounced improvements in aberrations and corneal shape as in the CXL-Plus group. However, the refraction of keratoconus patients can be challenging with notoriously variable results [26] and therefore does not represent an exact science in the narrower sense. In terms of percentages of lines gained or lost, CXL-Plus showed superior outcomes for UDVA (higher percentage of lines gained, lower percentage of lines lost), but slightly inferior results for BSCVA.

Investigating the reasons for visual loss, two main causes are conceivable: aberrations and opacity. If the total preoperative HOA are lower than the aberrations that are attributable to the anterior surface then the aberrations of the anterior surface may compensate, in part, for the posterior aberrations [1]. In regularizing the anterior surface, there is a risk of increasing overall aberrations, similar to the induction of HOA in keratoconic corneas fitted with rigid gas-permeable contact lenses [27]. However, we were able to exclude the corresponding risk situation for all patients with documented visual loss, with corneal opacity remaining as a possible cause. Virtually all studies on CXL report some degree of haze [28] and so does the present work: within the central 2 mm and the adjacent ring up to 6 mm, both groups showed an increase from just under twenty grayscale units to a mid-twenties range, where they also remained 24 months postoperatively. The densitometry values did not show a statistically significant difference between the groups at any point in time. However, we were unable to find a consistent pattern of densitometry values among patients who had lost two or more lines at the 12- or 24-month follow-up in this study. As a direct consequence for clinical practice, we stepped up our efforts to support epithelial healing by administering autologous serum eye drops if needed. Furthermore, topical steroids are restarted to fight stromal inflammation and scar formation in case of increasing densitometry values even months after treatment.

Our observations with regard to improvements in visual function and HOA are in accordance with two earlier reports on CXL-Plus that used the same iVIS™ Suite platform but that were published before the advent of the semi-automated CCR treatment planning mode [5, 29]. In both reports, the optical zone of the ablations had to be manually confined to a region near the corneal apex to save stromal tissue and to reduce the invasiveness of the treatments. In the present study, however, the software automatically calculated the most feasible regularization with the lowest corneal tissue consumption. The ablations were based exclusively on tomographic data and relieved the clinician of having to decide on a target refraction. Notably, the 50% of patients who gained 3 or more lines of UDVA compared favorably to alternative PRK strategies of varying complexity, such as conventional PRK with depth restriction [30], conventional PRK with variable degrees of cylinder correction [31], topography-guided PRK with variable spherocylindrical corrections [32], or topography-guided PRK with varying amounts of customization [6].

The present study has some limitations, which are partly due to the retrospective design. First, a formal assessment of the quality of vision which was not part of the routine clinical work-up. The main goal of the combined procedure was not only stabilization of ectasia, but also the best possible improvement of visual function. The optical prerequisites for this were demonstrably created with the reduction of the HOA. However, we did not use a standardized quality of life-visual function questionnaire to evaluate halo and glare nor did we assess the contrast sensitivity to determine the quality of visual function.

Second, not all patients were followed up at 24 months; if the patient’s condition was favorable and stable, the follow-up was often complete after 12 months, and the follow-up at 24 months was optional. For both groups, this may have meant that patients with less favorable clinical courses were preferentially seen at a 24-month follow-up, while patients with satisfactory outcomes may have opted not to have further follow-up. Interestingly, more patients of the CXL-Plus group appeared for the 24-month follow-up. We cannot exclude the possibility that clinicians were more interested in the long-term outcome of the combined procedure than in the outcome after CXL alone and that patients after CXL-Plus were therefore more likely to be encouraged to a 2-year follow-up. Possible disparities in the selection pattern may therefore have had an impact on group differences at that time point.

Third, patient allocation to one of the two groups was not random but was largely driven by the minimal corneal thickness that either allowed or disallowed the use of laser ablation. To prevent baseline differences, a probability matching approach was used with the intersection being formed between the CXL patients with the highest and the CXL-Plus patients with the lowest pachymetry values. This represented a negative selection within the CXL-Plus group in that the thickness of the stroma available for reshaping directly determined the possible extent of apex smoothing and thus limited the benefit of the combined treatment.

## Conclusions

The present study confirms previous preliminary studies showing that CXL-Plus improves corneal optics with respect to HOA. In light of the current evidence, CXL-Plus should be discussed as a treatment option with those keratoconus patients who wish to improve their UDVA and BSCVA rather than just preserving it. The automated calculation for excimer ablation proved to be useful, since clinicians did not have to enter a target refraction; further, the algorithm suggested an individualized, optimized regularization pattern for each cornea. This appears to be an important feature, as it requires less technical expertise than most of the current manual PRK methods and leads to a higher level of standardization of refractive treatments in combination with ectasia management, even when less experienced surgeons perform the procedure.

## Supplementary information

**Additional file 1 **Table A. Corneal cross-linking methods.

**Additional file 2.** Table B. Patient selection. For the CXL-Plus group, the potential study population was selected from all treated patients using the exclusion criteria. The same exclusion criteria were applied to all patients treated with CXL alone, beginning with patients who were treated the most recently, until a similarly large potential study population was obtained. A probability matching approach was then used to identify the final study groups with comparable baseline parameters with respect to UDVA, BSCVA, MRSE, corneal thickness, topographic cylinder, and Kmax.

## Data Availability

The datasets used and/or analyzed during the current study are available from the corresponding author on reasonable request.

## Abbreviations

BSCVA: Best spectacle-corrected visual acuity
CCR: Central corneal regularization
CIPTA™: Corneal Interactive Programmed Topographic Ablation
CXL: Corneal cross-linking
CXL-Plus: CXL with simultaneous PRK
HOA: Higher order aberrations
Kmax: Steepest keratometry value
LOA: Lower order aberrations
MRSE: Mean refractive spherical equivalent
PRK: Photorefractive keratectomy
RMS: Root mean square
UDVA: Uncorrected distance visual acuity
UV: Ultraviolet
