# Supplementary material for: Changes in higher order aberrations after central corneal regularization - a comparative two-year analysis of a semi-automated topography-guided photorefractive keratectomy combined with corneal cross-linking
Source: Eye Vis (Lond). 2020 Mar 3;7:10. doi: 10.1186/s40662-020-00179-2 (PMC7053096; doi:10.1186/s40662-020-00179-2)
Supplement: Supplementary file 1 — Additional file 1 Table A. Corneal cross-linking methods. [file 40662_2020_179_MOESM1_ESM.docx]

Additional file 1. Corneal cross-linking Methods.docx

Table A. Corneal cross-linking Methods

| **Parameter** | **Variables CXL-Plus group** | **Variables CXL group** |
| --- | --- | --- |
|  |  |  |
| Treatment target | Therapeutic refractive | Ectasia |
| Fluence (J/cm^2^) | 5.4 | 5.4 |
| Soak time and interval (min) | 30(q2) | 30(q2) |
| Intensity (mW) | 9 | 9 |
| Treatment time (min) | 10 | 10 |
| Treatment diameter (mm) | 9 | 9 |
| Epithelium status | Off (excimer laser) | Off (alcohol) |
| Chromophore | Riboflavin:  *i)* Peschke M®  *ii*) Peschke H® | Riboflavin:  *i)* Peschke M®  *ii*) Peschke H® |
| Chromophore carrier | *i*) HPMC  *ii*) - | *i*) HPMC  *ii*) - |
| Chromophore osmolarity | *i*) Iso-osmolar  *ii*) Hypo-osmolar | *i*) Iso-osmolar  *ii*) Hypo-osmolar |
| Chromophore concentration | 0.1% | 0.1% |
| Light source | CCL-365 vario (MLase AG) | CCL-365 vario (MLase AG) |
| Irradiation mode (interval) | Continuous | Continuous |
| Protocol modifications | Excimer laser | - |

HPMC = hydroxypropyl methylcellulose.
