# Supplementary material for: Changes in higher order aberrations after central corneal regularization - a comparative two-year analysis of a semi-automated topography-guided photorefractive keratectomy combined with corneal cross-linking
Source: Eye Vis (Lond). 2020 Mar 3;7:10. doi: 10.1186/s40662-020-00179-2 (PMC7053096; doi:10.1186/s40662-020-00179-2)
Supplement: Supplementary file 2 — Additional file 2. Table B. Patient selection. For the CXL-Plus group, the potential study population was selected from all treated patients using the exclusion criteria. The same exclusion criteria were applied to all patients treated with CXL alone, beginning with patients who were treated the most recently, until a similarly large potential study population was obtained. A probability matching approach was then used to identify the final study groups with comparable baseline parameters with respect to UDVA, BSCVA, MRSE, corneal thickness, topographic cylinder, and Kmax. [file 40662_2020_179_MOESM2_ESM.docx]

Additional file 2. Patient Selection.docx

Table B. Patient Selection

|  | **CXL-Plus group** | **CXL group** |
| --- | --- | --- |
| **Patients treated** | 204 | 636 |
| **Exclusion criteria** |  |  |
| Deviating CXL protocol | 13 | 22 |
| Age > 45 years | 24 | 47 |
| Indication for CXL other than keratoconus |  |  |
| Post-LASIK-ectasia | 1 | 14 |
| Post-Keratotomy Instability |  | 5 |
| Pellucid Marginal Degeneration | 1 | 19 |
| Keratoglobus | 1 | 3 |
| Infectious Keratitis |  | 16 |
| Ocular Comorbidity |  |  |
| Dry Eye |  | 3 |
| Fuchs`Endothelial Dystrophy | 1 |  |
| Map Dot Fingerprint Dystrophy | 1 |  |
| Corneal Scar | 1 | 4 |
| Others | 6 | 17 |
| Previous Penetrating Keratoplasty |  | 23 |
| Systemic Diagnosis |  |  |
| Trisomy 21 |  | 13 |
| Pregnancy |  | 1 |
| Diabetes Mellitus |  | 2 |
| Ankylosing Spondylitis | 1 |  |
| Wegener's Granulomatosis | 1 | 2 |
| Previous Renal Transplantation | 1 |  |
| Others |  | 4 |
| Follow Up |  |  |
| Period < two years | 19 | 139 |
| Loss Of follow-up | 6 | 20 |
| Incomplete data-sets | 31 | 32 |
| **Potential study population** | 96 | 96 |
| **Matched study population** | 28 | 28 |

For the CXL-Plus group, the potential study population was selected from all treated patients using the exclusion criteria. The same exclusion criteria were applied to all patients treated with CXL alone, beginning with patients who were treated the most recently, until a similarly large potential study population was obtained. A probability matching approach was then used to identify the final study groups with comparable baseline parameters with respect to UDVA, BSCVA, MRSE, corneal thickness, topographic cylinder, and Kmax.

CXL = corneal cross-linking, CXL-Plus = central corneal regularization combined with CXL, UDVA = uncorrected distance visual acuity, BSCVA = best spectacle-corrected visual acuity, MRSE = mean refractive spherical equivalent, Kmax = steepest keratometry value
